# Intermittent Dieting: Theoretical Considerations for the Athlete

**DOI:** 10.3390/sports7010022

**Published:** 2019-01-16

**Authors:** Jackson James Peos, Layne Eiseman Norton, Eric Russell Helms, Andrew Jacob Galpin, Paul Fournier

**Affiliations:** 1 The University of Western Australia (UWA), The School of Human Sciences, Crawley Campus, WA 6009, USA; paul.fournier@uwa.edu.au; 2 Biolayne LLC, 19401 Jacobs River Run, Lutz, FL 33559, USA; layne@biolayne.com; 3 Auckland University of Technology, Sports Performance Institute New Zealand (SPRINZ) at AUT Millennium, Auckland 0632, New Zealand; eric.helms@aut.ac.nz; 4 California State University, Biochemistry and Molecular Exercise Physiology Laboratory, Centre for Sport Performance, Fullerton, CA 92831, USA; agalpin@fullerton.edu

**Keywords:** intermittent energy restriction, diet—reducing, weight loss, caloric restriction, adaptive thermogenesis, composition—body, body weight maintenance

## Abstract

Athletes utilise numerous strategies to reduce body weight or body fat prior to competition. The traditional approach requires continuous energy restriction (CER) for the entire weight loss phase (typically days to weeks). However, there is some suggestion that intermittent energy restriction (IER), which involves alternating periods of energy restriction with periods of greater energy intake (referred to as ‘refeeds’ or ‘diet breaks’) may result in superior weight loss outcomes than CER. This may be due to refeed periods causing transitory restoration of energy balance. Some studies indicate that intermittent periods of energy balance during energy restriction attenuate some of the adaptive responses that resist the continuation of weight and fat loss. While IER—like CER—is known to effectively reduce body fat in non-athletes, evidence for effectiveness of IER in athletic populations is lacking. This review provides theoretical considerations for successful body composition adjustment using IER, with discussion of how the limited existing evidence can be cautiously applied in athlete practice.

## 1. Introduction

Athletes often undertake periods of weight loss in an attempt to reduce fat mass (FM) while retaining fat free mass (FFM). Although absolute body weight loss may be the primary outcome of concern for individuals with overweight or obesity, it is important that weight loss strategies adopted by athletes minimise losses of FFM, so as not to compromise training and performance [1]. Altering body composition in such a manner may be advantageous to the athlete for various biomechanical, aesthetic, and locomotive reasons, thereby increasing the likelihood of competitive success in a target weight-class (e.g., combat sports, weight lifting), weight-sensitive sports (e.g., endurance events, ski jumping), or aesthetically judged sports (e.g., gymnastics and bodybuilding) [2]. Previous literature indicates that athletes commonly reduce their absolute body weight by 5–10% over a number of months prior to competition [3,4], although more dramatic weight losses of ≥7% of body weight within 24 h have also been observed [5,6]. To achieve the desired weight loss (and to maintain sports performance), typically combinations of nutritional and exercise interventions are recommended for athletes [1]. Within this population, the most common nutritional weight loss strategy implemented is continuous energy restriction (CER), for the duration of the weight loss phase [7,8]. Specifically, CER involves reducing energy intake every day relative to weight maintenance energy requirements [9]. Despite being currently accepted as an evidence-based dietary intervention for weight loss, CER is accompanied by a number of behavioral, metabolic, and endocrine responses that collectively threaten dietary adherence, oppose the continuation of fat loss, and predispose the individual to weight and fat regain upon completion of the period of energy restriction (ER) [10,11,12,13,14,15]. Furthermore, it is well documented in athletes that ER, in conjunction with high training loads, can lead to loss of FFM and decreased performance via reductions in muscle strength, reflexes, and glycogen stores and increased irritability [2,16,17,18]. Elite athletes competing at a reduced body mass in weight-class and aesthetic sports also experience increased risk of injuries and chronic fatigue, and impaired immune function, which can lead to more frequent episodes of illness [19]. In these sports, failure to reach target body weight or body composition in the days or weeks prior to contest through the use of ineffective or suboptimal dietary strategies may cause athletes to depend on more rapid weight loss techniques that could jeopardise performance and possibly be dangerous. Such techniques include acute “water weight” loss, a practice that often involves severe dehydration via restriction of fluid intake, and actively pursuing sweating through exercise (often in combination with “sweat suits”) or the use of saunas and hot baths [20]. Dehydration is known to adversely affect athletic performance by reducing body water, electrolytes, and glycogen, which alter a number of physiological processes including metabolism, the regulation of body temperature, and cardiovascular function [7,20]. An additional challenge that athletes face when embarking on weight loss is the multitude of diet subtypes, the propagation of unfounded fad diets by the media, as well as conflicting nutritional research, all of which contribute to confusion regarding optimal manipulation of dietary variables for athlete weight reduction [21]. As such, there is an inherent need to review current and novel dietary strategies as a means of providing athletes with sound, evidence-based guidelines that facilitate the realisation of their body composition goals, without jeopardising health or performance.

Intermittent energy restriction (IER) is one nutritional strategy that has gained recent research attention [22,23,24,25,26,27] and which could potentially be of relevance to athletes wishing to reduce weight. IER involves alternating periods of ER with periods of greater energy intake (often referred to as ‘refeed’ periods or ‘diet breaks’), within the weight loss phase. Of note, these ER and refeed periods have also been referred to as ‘fast’ and ‘feed’ phases in some previous publications [9]. The proposed goal of implementing refeeds during periods of ER is to briefly stimulate the release of some regulatory hormones that play a positive role on fat loss and satiety and increase metabolic rate [2]. While a conceivable metabolic and hormonal model to attenuate ER-induced adaptations through the use of an IER regime exists, recent literature has not been definitive. The concept of integrating periods of greater energy intake within a weight loss phase received research attention following work by Wing and Jeffery [28]. Investigators examined the effects of disturbing the momentum of weight loss in an attempt to induce dietary relapses during a 14-week weight loss program. Surprisingly, prescribed diet breaks did not lead to a backsliding of progress, with participants who adopted either a six-week diet break at Week 7 of the program or two-week diet breaks after Weeks 3, 6, and 9 of the program not demonstrating any less weight losses (at 0–5 months or 0–11 months) when compared to the control group who dieted continuously for the 14-week program. These findings caused some researchers to speculate that diet breaks or refeeds could encourage greater adherence to longer-term diets in individuals needing to lose significant amounts of body fat.

Previous research in the realm of IER has focused on populations with overweight or obesity [29]. However, the metabolic and training status of athletes is considerably different from that of people who are overweight or who are sedentary. Athletes are typically of a healthy body composition and undertake high levels of physical activity and consequently have high energy expenditures, as well as a low probability of experiencing metabolic diseases or preliminary states of diseases, which are often observed in populations that are overweight and inactive [30]. Therefore, it is possible that these factors could influence the response to IER. Additionally, it is conceivable that IER strategies could be more likely to benefit athletes (or lean people in general) as evidenced by leaner individuals demonstrating two- to three-fold greater protein losses [31], greater reductions in testosterone levels [32], and a higher proportion of weight loss from FFM during ER when compared to individuals with a BMI in the overweight or obese range [33]. To our knowledge, no published research is currently available on IER in athletes. Thus, the purpose of this review is to discuss the existing body of literature on IER, outline its potential as an alternative weight loss strategy for athletes, and set a platform for future investigation in athletes. This review will also utilise the available evidence to develop theoretical recommendations for athletes considering IER.

## 2. Methods

PubMed, MEDLINE, SPORTDiscus and CINAHL electronic databases were searched online. Each author was assigned a section of the manuscript to write specific to their area(s) of expertise. Authors performed searches for key words associated with their section(s) of the manuscript; intermittent energy restriction, continuous energy restriction, adaptive thermogenesis, adaptive responses to energy restriction, athlete weight loss, nutrition for body composition management, and nutrition for athletic performance were the selected topics. Long-term human studies with individuals of a healthy body weight comparing energy-matched IER and CER protocols were preferentially selected; however, given the paucity of such studies, studies using overweight individuals and studies using animal models were also selected. In addition, author names and reference lists were used for further search of the selected papers for related references. As this review is intended to serve as an evidence-based guide and the available data applicable to athletes is limited, a narrative review style was chosen.

## 3. Adaptive Responses to Energy Restriction

Before discussing the existing literature and potential benefits of IER, it is worthwhile to review the adaptive responses to energy restriction and how they affect body composition management.

### 3.1. Energy Expenditure

Resting energy expenditure (REE) typically constitutes 60–75% of total daily energy expenditure (TDEE) and is a function of body mass (particularly FFM, but also FM). Thus, weight loss causes a decline in REE via the loss of metabolically active tissue [2]. However, weight-loss-induced reductions in REE—which occur in both lean and obese individuals—exceed that which is predicted by decreases in FFM and FM alone [34,35]. Termed “adaptive thermogenesis” (AT), this metabolic alteration successively minimises the degree of energy deficit achieved by ER. AT can partially explain why weight loss plateaus are encountered despite continued intake of a diet with less energy than before weight loss, and likely contributes to the restoration of baseline body weight upon termination of an energy-restricted state [35,36,37]. AT has been studied frequently in overweight and obese populations, but not in athletes. One investigation reported that AT explained ~50% of the less-than-expected weight loss in obese women undergoing ~14 weeks of 4200 kJ per day of ER [38]. A recent review highlighted the potential impact of AT by concluding that weight loss strategies are only transiently effective, as the majority of overweight or obese individuals were not able to achieve and maintain a 10% reduction in absolute body weight over 12 months [12]. A meta-analysis also showed that in individuals who completed structured weight loss programs, over a third of the weight lost returned within the first year, with the majority of the rest gained back within 3–5 years [39]. Furthermore, in men with a healthy body weight, 24 weeks of ER (50% of energy requirements for weight maintenance) caused a 40% reduction in baseline energy expenditure. Weight loss accounted for 25% of this reduction and AT explained the remaining 15% [40].

**Figure 1:**
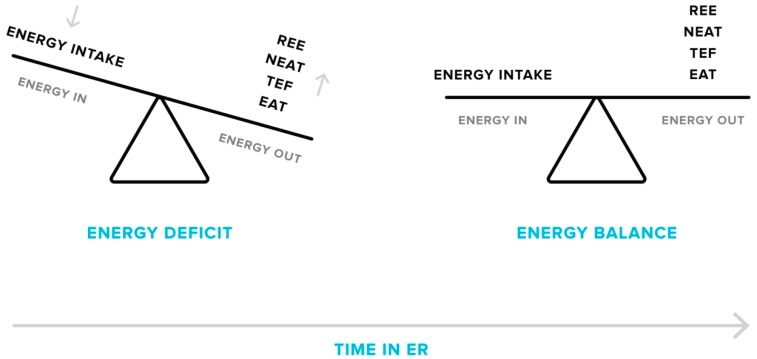
Adaptive responses in energy expenditure during energy restriction (ER). Over the course of a weight loss phase, total daily energy expenditure will decrease as a consequence of declines in resting energy expenditure (REE), non-exercise (NEAT) and exercise activity thermogenesis (EAT), and the thermic effect of feeding (TEF). This results in a lessening of the energy deficit, which can cause plateaus in weight loss if energy intake matches the new level of energy output. Plateaus may only be overcome by a further reduction in energy intake or an increase in activity levels.

Along with decreases in the resting component of TDEE, non-resting components, including exercise activity thermogenesis (EAT; energy expended for sports-like exercise and physical training) and non-exercise activity thermogenesis (NEAT; energy expended for activities that do not include sleeping, eating or sports-like exercise), also decline as reduced body mass demands less energy to complete certain activities, particularly when weight-bearing locomotion is involved [36,41,42,43,44,45]. One cross-sectional study found an 18% lower TDEE in people with obesity after a body weight loss of 23% when compared to individuals with the same body weight who had never dieted [42]. It was observed that the decrease in TDEE was largely due to reductions in non-resting components, accounting for 71% of the observed difference. Finally, the thermic effect of feeding (TEF) is lowered by consuming less energy overall during ER [41,46]. Reductions in TEF accounted for 18% of the fall in TDEE in people with obesity after weight loss [41]. Thus, the evidence suggests that ER and the associated weight loss cause a metabolic downshift in all components of TDEE (Figure 1), which could significantly influence long-term body weight management.

### 3.2. Endocrine Responses

A number of endocrine responses also accompany ER, including variations in thyroid, appetite-regulating, and steroid hormones that collectively influence energy expenditure, body composition, and satiety. Thyroid hormones play a prominent role in regulating energy expenditure whereby decreases in circulating or tissue levels of active forms reduce thermogenesis and REE [47]. Previous research suggests that up to 30% of REE is determined by thyroid hormones [48]. In terms of body composition, clinical observations in humans show that hypothyroidism can lead to a metabolic shift culminating in an increase in FM in combination with reduced FFM [49]. As reviewed previously, ER is associated with decreased thyroid hormone secretion (T_3_ and T_4_) in lean healthy men and women [50]. A similar finding was also observed in a case study of a competitive athlete undergoing ER in preparation for a bodybuilding contest [51]. Additionally, in overweight or obese people, moderate ER has been shown to reduce overall hypothalamo-pituitary-thyroid axis function, causing a decline in circulating or tissue concentrations of active thyroid hormones, which is associated with a significant decrease in energy expenditure [52]. It is therefore possible that the inhibition of normal thyroid function in athletes undergoing ER may negatively influence the propensity for long-term weight/fat loss and increase the likelihood of loss of FFM and weight/fat regain.

Two well-known appetite-regulating hormones are leptin and ghrelin, which work in opposition of each other in this context. Leptin, a hormone largely synthesized in adipocytes, responds to energy availability such that low levels increase appetite while high concentrations yield the opposite response [53]. Leptin has also been shown to increase energy expenditure via effects on the hypothalamus [54]. Worthy of note, reductions in circulating leptin are frequently observed in ER studies of lean competitive athletes [13,14,51,55]. In men with a healthy body composition undergoing three weeks of ER (50% of energy requirements for weight maintenance), plasma leptin was reduced by 44% [56]. Another trial found that four days of ER (~60 kJ/kg of FFM per day) reduced circulating leptin by 53–56% in regularly exercising men [57]. The above evidence suggests leptin not only responds to overall adiposity but also short-term nutrient flux. Pharmacological administration of leptin can reverse some of the adaptive responses to ER—namely—reductions in REE, skeletal muscle work efficiency, and circulating thyroid hormone concentrations [58], yet further discussion of such interventions is beyond the scope of this paper.

In general opposition of leptin, the orexigenic hormone ghrelin reflects acute and chronic feeding states by increasing the drive to eat during periods of fasting and low energy intake [59]. As reviewed previously, since plasma ghrelin levels are dependent on recent energy intake, this hormone plays an important role in regulating hunger and meal initiation [60]. One study reported that plasma ghrelin-like immunoreactivity increased 31% after 12 h of fasting and was reduced by 22% immediately after a meal [59]. Leptin and ghrelin (absolute amounts and ratios) were significantly correlated with FM loss and changes in REE at both baseline and following 12 weeks of ER in obese women [61]. Therefore, lower leptin and higher ghrelin appear to oppose FM loss and may be viable biomarkers for predicting the magnitude of metabolic adaptations to ER, yet more research is needed to confirm. Decreased leptin and increased ghrelin in response to ER may oppose potential weight loss by significantly altering appetite (which could cause compensatory eating) or by reducing energy expenditure.

Insulin, which is an additional adiposity signalling hormone, responds to energy availability in a similar manner to leptin and plays an important role in preventing muscle protein degradation [62]. In humans—with either a healthy body weight or with obesity—significant reductions in fasting insulin concentrations have been reported during ER, potentially threatening maintenance of FFM [63,64,65,66]. Reduced insulin levels during ER are disproportionately low when compared to equal relative FM at a stable body weight, indicating a physiological shift acting to correct the state of energy depletion and favour weight regain [67]. Athletes recovering from strenuous exercise also require depleted fuel stores to be replenished, primarily through the insulin-mediated uptake of glucose in muscle [68]. Therefore, reduced insulin levels as a consequence of ER may impact the restoration of muscle glycogen stores. The importance of sufficient muscle glycogen content for the athlete is further discussed in Section 6.2.3.

Reduced activity of the reproductive and somatotropic axes is also a possibility during dynamic ER [49]. For instance, 8 weeks of ER (via heavy exercise and reduced energy intake) significantly reduced serum testosterone concentrations in healthy lean men [69]. Testosterone may be of particular interest to athletes due to its role in stimulating muscle protein anabolism leading to accumulation (and maintenance) of muscle mass [62]. However, several studies also confirmed a significant inverse correlation between reductions in FM and circulating testosterone levels [70]. Interestingly, some studies show that ER in men with a BMI in the overweight range either increases or has no impact on circulating free or total testosterone levels [71]. Therefore, reductions in testosterone during ER may only be of concern to individuals with a lean body composition. While reduced circulating concentrations of insulin-like growth factor-1 (IGF-1) has been observed in some cases of ER [72], it does not appear that growth hormone (GH) is significantly disrupted, as evidenced by no change in GH levels after 6 months of ER (25% below weight maintenance requirements) [73].

In contrast to testosterone, serum increases in the glucocorticoid cortisol causes protein catabolism in healthy subjects [74]. Furthermore, a rodent trial showed glucocorticoid administration inhibited the potent energy dissipation and satiating effects of leptin, thereby suggesting glucocorticoids play a counterregulatory role on leptin action and contribute to leptin resistance [75]. Chronic hypercortisolism regularly occurs in highly trained athletes [76]. Additionally, salivary cortisol levels were elevated in male and female competitive gymnasts when compared to age-matched non-athletes [77]. While psychological stress likely played a role in the observed cortisol elevations, ER was a probable significant contributor. This is further evidenced by significant increases in serum cortisol in highly active lean men completing 8 weeks of ER resulting in weight loss [69]. As discussed in a narrative review, glucocorticoids such as cortisol also promote FM accretion [49]. Therefore, increases in cortisol output during ER may impede athlete weight loss efforts by promoting the conservation of FM and loss of FFM and by inhibiting the actions of leptin.

**Figure 2:**
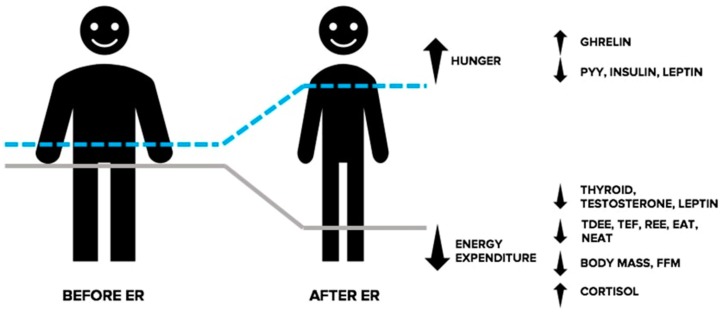
Adaptive responses in the endocrine system during energy restriction (ER). In response to ER, the resulting energy deficit and corresponding weight loss causes an increase in the drive to eat and reduced energy expenditure, collectively making the continuation of weight loss more challenging. Changes in circulating levels of orexigenic and anorexigenic hormones communicate a nutrient deprivation signal to the brain, causing stimulation of appetite, and a decrease in feelings of satiation. Furthermore, ER causes a shift in circulating levels of hormones involved with the regulation of thermogenesis and energy expenditure. Changes in these hormones indicate a physiological shift directed at correcting the state of energy deprivation and favouring weight regain. EAT: exercise activity thermogenesis; FFM: fat free mass; NEAT: non-exercise activity thermogenesis; PYY: peptide YY; REE: resting energy expenditure.

While not an exhaustive list of endocrine responses associated with ER, the above text illustrates a shift in the hormonal milieu in response to energy deprivation that collectively promotes reductions in energy expenditure and an increased drive to eat and threatens the retention of FFM (See Figure 2). For a more comprehensive explanation of the endocrine responses accompanying ER, we direct the reader to the following reviews [49,52,78].

### 3.3. Adaptations in Adipose Tissues

**Figure 3:**
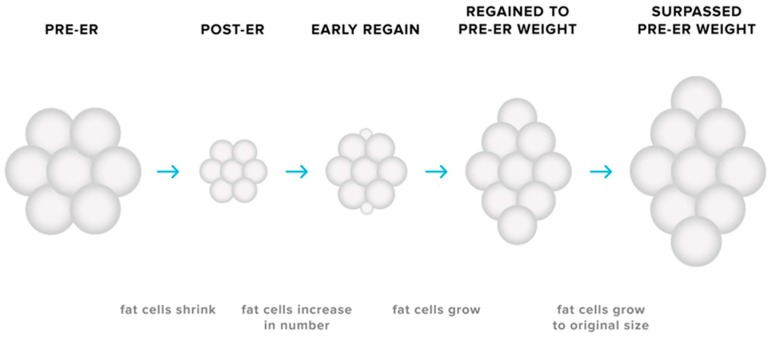
Adaptive responses in adipose tissues during energy restriction (ER). ER causes a decrease in the size of adipocytes, with no discernible change in adipocyte number in the adipose depot. Due to the modification of the metabolic profile of these smaller adipocytes, the potential for storage of triglyceride increases, subsequently making the maintenance of lost weight more challenging. The possibility of adipocyte hyperplasia early in the weight regain period may also increase the likelihood of weight-reduced individuals surpassing their pre-energy-restriction body weight.

The cellularity and metabolic characteristics of adipose tissues may contribute to the biological tendency for weight regain after weight loss. Reductions in body fat is accompanied by a decrease in the size of adipocytes as energy stores are mobilised [79,80], yet no discernible change in the number of adipocytes present in adipose tissue is observed [79,80,81,82]. Smaller adipocytes secrete less leptin for a given FM [12], therefore implying that the reduction in adipocyte size—as well as total FM—may contribute to the ER-induced decline in circulating levels of leptin. Furthermore, smaller adipocytes exhibit higher glucose uptake rates [83,84], a greater expression of genes favouring energy storage [85,86,87], and reduced lipolysis [12] compared to larger adipocytes. As reductions in adipocyte size has also been linked to remodelling of the extracellular matrix to accommodate this change in propensity for nutrient storage, it has been proposed that weight loss induces cellular stress that modifies the metabolic profile of adipocytes [88]. Conceivably, such a change would only be relieved via increased storage of lipid [89]. While the number of adipocytes remains relatively constant during adulthood [90], there are circumstances in which adipocyte number may increase. Previously published research in rodents demonstrates that adipocyte hyperplasia can occur in the early stages of weight regain after weight loss, resulting in a greater number of adipocytes in adipose tissue depots and thus a greater potential for storage of lipid [91]. Hyperplasia in adipose tissue could conceivably amplify the likelihood of rapid weight regain not only by increasing the size of the depot but also through the creation of small adipocytes with an enhanced capacity for uptake and storage of ingested nutrients (see Figure 3). It has been speculated that this phenomenon may partially explain situations in which pre-ER body weight is surpassed during the post-weight loss period [12,79,80,86]. Thus, the adaptations in adipose tissue—in combination with changes in energy expenditure and the endocrine milieu—can present a substantial challenge not only for successful weight loss but also for the maintenance of the reduced body weight.

## 4. Intermittent Energy Restriction: An Overview of Effectiveness

### 4.1. Intermittent Fasting

IER lacks clear definition, acting as an umbrella term encompassing a variety of dietary protocols that utilise different energy prescriptions during ER and refeeds as well as the length of these periods. The most common IER used in clinical trials is often referred to as ‘intermittent fasting’ (IF) [29]. IF has been defined previously as severe reduction of energy intake on 1–7 days, followed by a relatively greater energy intake on refeed days [29,92]. There may or may not be restrictions placed on the quantities and types of foods and beverages consumed during these refeed periods [27]. It is important to note that IF is one form of IER but has also been used to describe various other dietary interventions including time-restricted feeding approaches (such as the 8 h feeding window method) [93]. Alternate-day fasting is a sub-type of IF that involves partial or complete restriction of energy intake for 24 h, followed by a refeed day where food is consumed ad libitum for 24 h [9,94,95,96]. Another sub-type of IF—known as the 5:2 (or ‘fast’) diet—alternates two days of severe ER with five refeed days per week [27,97,98]. The available evidence indicates that IF is not superior to CER as a weight loss strategy. In a recent review of 13 randomised comparisons of CER and IF in overweight individuals, all studies demonstrated comparable reductions in body weight where overall energy intake was matched [24]. In another recent review investigating the effectiveness of randomised controlled trials of IER compared with CER in overweight and obese adults, the authors concluded that IER involving ER on at least one day per week but no greater than seven days were equally effective as CER for short-term weight management [99]. Furthermore, the majority of trials reporting body composition outcomes have shown equal efficacy for reducing FM, visceral fat stores, and waist circumference when IF regimes were compared to CER in adults with overweight and obesity [24,92,97]. While the mass of literature has failed to establish a benefit to short-term IER regimes implementing ER on 1–7 days per week, greater weight loss efficiency (weight lost per unit of ER) was observed in mice with diet-induced obesity following an IER diet that involved 5–6 days of ER (82% of ad libitum chow-fed control) followed by ad libitum refeeding for 1–3 days, compared to CER (82% of ad libitum chow-fed control), after 12 weeks (0.042 ± 0.007 versus 0.018 ± 0.001 g/kJ, respectively) [27]. Despite the IER group eating significantly more than the CER group (91 versus 82% of control intake, respectively), the same magnitude of weight loss was achieved in both groups. Although these findings have not been replicated in published controlled human trials, potential benefits of IER have been recently demonstrated in preliminary research presented as a conference abstract [100,101]. An IER regime alternating five days of ER with twice-weekly refeeds was superior to CER in preserving REE (−40 kcal·d^−1^, *p* = 0.410 versus −78 kcal·d^−1^, *p* = 0.017) and FFM (−0.4 kg, *p* = 0.460 versus −1.3 kg, *p* = 0.001)—while causing equivalent FM loss (2.8 versus 2.3 kg)—among resistance-trained athletes completing seven weeks of ER (energy intake 25% below weight maintenance energy requirements).

In one review, it was reported that alternate-day fasting caused less loss of FFM, with equivalent FM loss when compared to CER in adults with overweight and obesity [9]. This caused some to suggest that IF may be a more effective strategy than CER for mitigating FFM losses during ER. However, a recent randomised controlled trial involving 100 adults with overweight or obesity randomised to alternate day fasting or CER for 24 weeks showed similar improvements in the FFM-to-total-mass ratio between groups [25]. Additionally, a recent systematic review of 31 publications comparing IF with CER in humans of any age or BMI failed to demonstrate superior FFM retention using IF [27].

One proposed benefit of adopting an IER regime over CER is that refeed periods may provide a mental break from extended periods of ER, leading to improved long-term adherence to the dietary schedule. In one randomised controlled trial, participants were randomly allocated to a role-playing scenario that simulated either seven days of an IER model (six days of 1300 kcal/d followed by one day of 2700 kcal/d refeed) or CER (seven days of 1500 kcal/d) [102]. While both simulated conditions were energetically matched (10,500 kcal per week), participants in the IER group demonstrated a higher-than-expected self-regulatory ability (they were expected to experience more positive affect during the diet phase) and generated more strategies to overcome dietary temptations than participants in the CER group. However, in a recent systematic review comparing short-term IER protocols (≤7 days cumulative ER) with CER, dropout rates varied between 0 and 40% and were comparable between both groups, and to previous reports within other CER studies [24]. This suggests long-term adherence to short-term IER protocols may be similar to CER, presenting a viable—albeit not superior—alternative for individuals who find sustained daily ER difficult.

### 4.2. IER Regimes Involving Longer Periods of ER

In contrast to the above findings, some recent randomised controlled trials show better weight and fat loss with certain types of IER that incorporated longer-term periods of ER and refeeds (i.e., ≥7 days of cumulative ER alternated with a lesser degree of prescribed dietary restriction, or no prescribed dietary restriction), when compared to CER. One possible reason for the apparently greater weight and fat loss, is that these protocols may mitigate some of the adaptive responses to ER that oppose ongoing weight and fat reduction. A six-week diet involving CER (45% of weight maintenance energy requirements) was compared with a six-week diet involving IER that cycled 11 days of ER (55% of weight maintenance energy requirements) with 3 days of ad libitum feeding in 74 women with obesity [98]. There was significantly greater weight loss at four weeks after completion of the diet in women in the IER than in the CER group (5.8 ± 1.2 versus 3.4 ± 1.4% of initial body weight, respectively). Moreover, REE was maintained at higher levels in the IER than in the CER group. Perhaps the most convincing evidence for adopting a long-form IER protocol (over CER) can be found in findings from the recent “MATADOR” study. In this randomised controlled trial, 16 weeks of CER (67% of weight maintenance energy requirements) was compared with 16 weeks of ER applied intermittently as eight two-week blocks of ER (67% of weight maintenance energy requirements) alternating with seven two-week blocks of EB (where energy intake is matched to energy requirements for weight maintenance) in 51 men with obesity [22]. Significantly greater weight loss (14.1 ± 5.6 versus 9.1 ± 2.9 kg, *p* < 0.001) and FM loss (12.3 ± 4.8 versus 8.0 ± 4.2 kg, *p* < 0.01), as well as less compensatory reduction in REE (−360 ± 500 versus −750 ± 500 kJ·d^−1^, *p* < 0.05) and better maintenance of lost weight at a six-month follow-up (−11.1 ± 7.4 versus −3.0 ± 4.4 kg, *p* = 0.001), were observed in the IER compared to the CER group. Furthermore, FFM loss was the same in both groups despite greater FM loss in the IER group. In a third randomised controlled trial, equal weight loss was achieved among overweight women following either 8 weeks of CER (5500 kJ ER per day) or 8 weeks of IER administered as one week of ER (5500 kJ ER per day) alternating with one week of the participants’ habitual diet (no prescribed ER) (−3.2 ± 2.1 kg CER, −2.0 ± 1.9 kg IER) [103]. While findings could be interpreted as a lack of superiority to this long-form IER protocol due to equivalent weight loss, it is important to consider that the IER group spent less time in ER compared to the CER group (4 weeks in IER versus 8 weeks in CER), resulting in greater weight loss efficiency.

Findings from these trials indicate that these forms of IER attenuated the adaptive response to ER, at least with respect to reductions in REE. Strategies—such as intermittent refeed periods—that potentially attenuate reductions in energy expenditure are important, as literature demonstrates people who regained >30% of weight lost after ER showed reduced energy expenditures when compared to those who remained weight-stable (within <±20% of weight change) [104]. Furthermore, the estimated risk of gaining >7.5 kg in body weight over a two-year longitudinal study increased fourfold in people with low 24 h energy expenditures compared to people with high 24 h energy expenditures [105]. Adjusted REE was also found to predict the gain in body weight over a four-year follow-up period [105].

**Figure 4:**
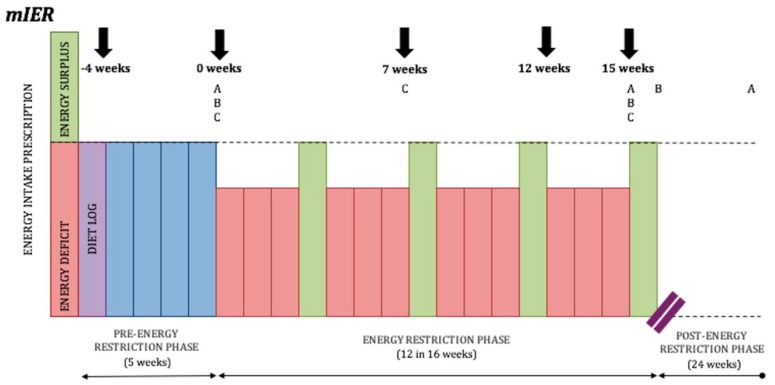
Long-form IER protocol designed by Peos and colleagues. (**A**) Fat mass, fat free mass, and body weight measured at 0 weeks, 15 weeks, and at 6 months in the moderate intermittent energy restriction group (mIER); (**B**) muscle performance, resting energy expenditure, a drive to eat, and levels of appetite-regulating hormones measured at 0 weeks, 15 weeks, and 16 weeks; (**C**) mood states, diet acceptability, physical activity, and sleep quality measured at 0 weeks, 7 weeks, and 15 weeks.

Although the above long-form IER protocols display some inherent benefits over CER, in a recent systematic review and meta-analysis—which did not include the above studies—five randomised controlled trials of IER incorporating ≥7 days of cumulative ER were compared with CER for the treatment of overweight and obesity in adults [23]. Meta-analysis demonstrated no significant difference in weight loss at post-intervention (weighted mean difference: −1.36 [−3.23, 0.51], *p* = 0.15) or at follow-up (weighted mean difference: −0.82 [−3.76, 2.11], *p* = 0.58). To help clarify the disparity in the literature, Peos and colleagues will be the first to examine a long-form IER regime alternating three-week blocks of ER with one-week blocks of EB in resistance-trained athletes [106] (See Figure 4).

While attenuation of the decline in REE has been observed with some IER protocols when compared to CER, it remains to be seen whether these dietary regimes also result in minimisation of some of the other adaptive responses associated with ER. Under some circumstances, a number of the adverse metabolic and hormonal outcomes associated with weight loss have been reported to be quickly reversed upon restoration of EB, namely normalisation of fasting and postprandial energy expenditure, and circulating levels of leptin and thyroid [107,108,109,110,111,112,113]. In one trial among postmenopausal women, following 10 days of ER (3350 kJ per day), reductions in body composition-adjusted REE and circulating levels of thyroid hormones were returned to baseline after 10 days in EB [114]. Furthermore, in overweight women following a very-low-energy diet for 28 days, suppressed serum thyroid parameters returned to baseline concentrations following one week of increased energy intake (4200 kJ) [115]. Thus—as previously discussed in a narrative review—a number of the undesirable consequences associated with weight loss are related to the ER itself, not to the weight loss per se [12].

There is evidence that levels of leptin [116] and thyroid hormones [117] can be temporarily increased following short-term overfeeding. In one trial, the subsequent elevation in leptin following overfeeding resulted in a 7% increase in TDEE [118]. These findings have caused speculation among some athletes and coaches that refeed periods may stimulate an increase in circulating levels of these regulatory hormones leading to temporarily inflated metabolic output and reversal of adaptive mechanisms associated with ER. However, it remains to be seen whether the same stimulatory effect on leptin release and thyroid activity can be achieved with controlled refeeding during IER where participants temporarily increase energy intake to levels for EB, as opposed to overfeeding. If this was indeed the case, the stimulatory effects on energy expenditure and fat loss via increased thyroid and leptin output, in conjunction with leptin’s influence on satiety, would likely decrease the drive to eat—enabling better dietary adherence—and facilitate greater weight loss efficiency. Recently, in a series of in-depth interviews with seven experienced male competitive bodybuilders, participants frequently reported the implementation of refeeds during contest preparation [119]. According to the participants, the purpose of refeeds was to “prevent downgrades in energy expenditure, replenish intramuscular glycogen stores, and provide mental refreshment.” Some participants claimed better fat loss, muscle retention, and less reduction in energy expenditure when using refeeds during pre-contest weight loss, compared to weight loss without refeeds. However, it is unknown how the participants were able to perceive such differences.

### 4.3. Limitations of the Existing Literature

The current body of research suggests IER may be offered as a viable, albeit not superior, alternative to CER for management of body composition. However, several limitations persist. Firstly, many IER protocols are collectively grouped together in systematic reviews and meta-analyses despite substantial heterogeneity between dietary regimes, suggesting a notion that all IER models are similar in design and efficacy. Therefore, it is difficult to determine whether equivalent findings in many of these reviews were due to certain IER manipulations or IER itself. An additional limitation is the inclusion of IER regimes that still prescribed an energy deficit (albeit minimal) during refeed periods, or trials where participants were instructed to eat ad libitum on occasion, but ate far less than expected and thus did not attain EB. As previously mentioned, the attenuation of adverse responses to ER using long-form IER protocols appears to be dependent on the restoration of true EB [107,108]. Hence, comparable findings between CER and IER protocols that did not establish EB during refeed periods should be expected. Another concern for a number of trials included in the reviews is whether there was sufficient statistical power to detect sensitive differences in the loss of FM and FFM between diet arms.

## 5. Practical Considerations: Intermittent Energy Restriction for Athletes

As the majority of above-mentioned research is limited to overweight and obese populations, it is unknown whether athletes respond to IER in a similar fashion. Athletes commonly need to reduce weight for sport purposes, so the following will take use of the available literature to discuss some of the practical strategies athletes considering IER could use to engage in the most evidence-based weight loss approach. We will discuss implications of incorporating resistance exercise during IER and considerations for the size of the relative energy deficit, the duration of weight loss, and dietary composition. While randomised controlled trials exploring IER in active individuals of a healthy body composition are scarce, there is evidence of athletes already adopting this dietary strategy [2,120,121]. However, there is a lack of consensus among coaches and dieticians on how to successfully employ IER in athletes, with many IER protocols being based on speculation or anecdotal evidence.

### 5.1. Resistance Exercise

As FFM is a critical predictor of REE, minimising losses may enhance long-term weight loss success and prevent rapid weight regain by abating metabolic downturn. A commonly cited ‘rule’ suggests that ~25% of weight loss will be FFM, with the remaining 75% FM [33]. Yet this is inappropriate, often underestimating the proportion of FFM lost during weight loss [10]. Previous findings report ~40% of weight loss was accounted for by FFM in normal-weight active army rangers after losing 10 kg over 8 weeks [122]. In lightweight rowers, 6% body weight loss over 8 weeks resulted in 50% attributed to FFM loss [123]. However, evidence suggests that resistance exercise may preserve FFM during weight loss in both men and women with either healthy weight or obesity, completing moderate (~2000 kJ ER per day) or severe (~3200 kJ very-low-calorie-diet) ER [120,124,125,126]. Thus, resistance exercise appears to be a worthwhile strategy for athletes to implement during IER. In keeping with this stand, elite athletes undergoing moderate ER (0.7–1.4% absolute body weight loss per week) who concomitantly undertook four intense resistance exercise sessions per week were shown to retain their FFM [127]. Additionally, moderate weight loss in female athletes performing weekly aerobic and resistance exercise did not cause reduction in their FFM after four weeks, despite the participants achieving absolute body weight losses of 0.5 kg per week [128]. However, preserving FFM during ER appears to be extremely difficult for very lean male and female athletes, regardless of intense resistance exercise [129,130,131]. In elite lean male bodybuilders (9.1% body fat prior to beginning ER), nine weeks of ER reduced percentage body fat to 5.0% but was accompanied by a significant loss of FFM from 90.60 to 88.14 kg [130]. Furthermore, in five lean athletic females and five female competitive bodybuilders, 12 weeks of ER in preparation for a contest resulted in a 5.80 kg loss of body weight, with 23.8% of this weight loss accounted for by reductions in FFM [131]. With the evidence considered, it appears that adiposity has a protective effect against loss of FFM during ER and should therefore be of greater concern for lean individuals considering weight loss interventions. As a final note concerning resistance exercise prescription—IER may have particular application to training athletes by allowing the coordination of refeed periods with key performance outcome-focused training sessions/blocks. Such a tactic would provide optimal nutritional support for these sessions while potentially negating the unwanted performance consequences of sustained daily ER.

### 5.2. Avoiding Severe Energy Restriction

A limitation of some IER protocols is that the level of restriction is severe, particularly with IF. In a recent review of weight loss composition [33] and FFM–FM interrelationships in humans [132], it was recognised that greater degrees of ER are often accompanied by larger relative FFM losses. In addition to poorer FFM retention, severe ER has previously been associated with less weight loss efficiency, greater metabolic fluctuation, and excessive hyperphagia when compared to more moderate ER in athletes, and in overweight individuals [127,133,134,135,136]. Furthermore, adhering to intermittent severe reductions in energy intake may prove difficult for some people, as evidenced in a systematic review which identified an increase in appetite and irritability in individuals assigned to IF [29]. In most instances, it would seem wise for athletes to avoid severe ER due to potential adverse health and performance outcomes. As recognised in previous reviews and a recent consensus statement from the International Olympic Committee [1,2,18,127], these outcomes include reductions in muscle strength, glycogen stores and reflexes, and increased risk of injury due to fatigue. Published scientific trials also demonstrate that gradual weight loss rates are superior to aggressive ones for athletes. In female athletes, 0.5 kg of weight loss per week was preferable over 1 kg per week after 4 weeks of ER, with a 30% greater reduction in testosterone levels and a 5% decrease in bench press strength in the faster weight loss group [128]. Additionally, comparing weekly weight loss rates of 0.7% to 1.4% (of absolute body weight) in elite athletes displayed greater fat loss (31% ± 3% versus 21 ± 4%) and an increase in FFM in the slower group versus no change in the faster group (2.1% ± 0.4% versus −0.2% ± 0.7%, respectively) [127]. As previously discussed in a recent review, it seems wise to tailor energy deficits during ER to cause moderate weight loss of 0.5–1% of body weight per week, to minimise FFM loss and performance decrement [137]. With the evidence considered, an athlete considering IER should be encouraged to adopt moderate, as opposed to severe, ER. As a practical example of moderate ER, this could involve a maximum of 35% restriction of energy intake relative to weight maintenance energy requirements [138].

### 5.3. Duration of Weight Loss and Refeeds

Recent findings demonstrated that a diet involving IER which cycled two weeks of moderate ER with two weeks in EB was superior to moderate CER in men with obesity, in terms of fat loss and maintenance of REE after 12 weeks of ER [22]. However, a caveat of this IER model is the considerably greater time required within the intervention in the IER group (30 weeks versus 16 weeks), despite both groups completing equal time in ER. Typically, athletes will reduce body weight for competition over 8–16 weeks [1], so the above IER protocol may seem unattractive to athletes by significantly extending the duration of the weight loss phase. A worthy question is, could the same increase in fat loss efficiency, and the attenuation of REE reduction be achieved if blocks of EB were less frequent and of less duration (hence reducing total intervention duration)? Such a result may increase the appeal of IER to the athletic community. Peos and colleagues aim to answer this question by investigating a three-week-ER–one-week-EB model [106].

As an additional consideration, it might be important that periods of EB implemented during IER are not too short, as available research on overweight adults suggests that the reversal of some of the compensatory responses to ER may require at least 7–14 days in EB [107,108,139,140]. In one trial, it was observed that reductions in REE consequent to ER in obese women could persist ≥8 weeks post-weight loss despite an increase in energy intake and weight stabilisation [141]. The persistence of some of the adaptive responses to ER beyond the weight loss period has also been observed in active people with a healthy body composition. Twenty-seven female fitness competitors completed four months of ER and successively reduced their FM by ~35–50% (DEXA, bioimpedance, skinfolds, *p* < 0.001) [142]. After a 3–4-month recovery period comprising an increase in energy intake to pre-diet values and concomitant reduction in aerobic exercise, testosterone and thyroid hormones (T_3_) had still not returned to baseline.

These findings suggest that ER awakens the body’s homeostatic defence system in a manner that is persistent, and not easily counteracted. Interestingly, short-term 24–48 h refeed periods are commonly implemented in athlete weight loss practice [2,100,101,119,143]. While available literature does not indicate that the complete resolution of the adaptive responses to ER is likely with such short-term bouts of refeeding, it is unknown whether this strategy could still provide partial normalisation to pre-diet values—and if so—what implications this may have on weight reduction efforts. Although the effects of short-term refeeding (24–48 h) has not been examined extensively in trials involving athletes, aggressive 24 h refeeding has been investigated in energy-restricted women with suppressed luteinizing hormone pulse frequency (−57% to 8.1 +/− 1.5 pulses per 24 h) and thyroid hormone levels (T_3_ −22% *p* < 0.005) [144]. Following one day of refeeding (~375 kJ per kg of FFM), the acute increase in energy intake was not sufficient to restore thyroid profiles or luteinizing hormone pulsatility. However, in a second randomised controlled trial, 48 h of refeeding after a three-day fast restored luteinizing hormone pulsatility in a group of women within 15% of their ideal body weight [145]. Furthermore, better maintenance of REE during weight loss was observed using weekly 48 h refeeds versus CER in a cohort of resistance trained athletes [100,101]. Therefore, the evidence suggests that not just the magnitude of energy intake but also the time spent within EB is important in achieving the reversal of some of the adaptive responses associated with ER.

## 6. Dietary Considerations: Intermittent Energy Restriction for Athletes

The effect of varying macronutrient ratios within a given energy quota has received considerable research attention. ER induces weight loss by imposing an energy deficit, regardless of whether a particular diet is geared toward manipulation of a certain macronutrient (e.g., low fat, low carbohydrate, and high protein) [21,146]. However, approaching weight loss in a solely “energy in/energy out” manner fails to consider the effects dietary composition may have on additional outcomes including the composition of weight lost, satiety, the thermic effect of feeding, and ease of compliance. As for micronutrition, athletes will typically not require vitamin and mineral supplementation while consuming adequate energy to maintain body weight from a variety of food sources. However, during ER (particularly severe ER), or if the diet consists largely of foods low in micronutrient density it may be necessary to consume a vitamin/mineral supplement to reach daily micronutrient requirements. For further reading on this topic, we direct the reader to [147].

### 6.1. Protein Intake

Protein increases satiety and has a higher thermic effect of feeding compared to carbohydrate or fat, so higher protein (>25% of energy intake) diets may be beneficial to weight management by increasing energy expenditure and improving compliance [148,149,150,151]. As the success of a weight loss diet is ultimately determined by adherence to a set intake, ease of compliance should be an important consideration. Reduced energy intake was reported in healthy individuals fed an ad libitum high-protein diet (30% of energy intake) versus an isocaloric lower protein diet (15% of energy intake), demonstrating the satiating effects of dietary protein [152]. High protein diets also exert stimulatory effects on muscle anabolism, reducing FFM losses during ER [152,153,154]. In resistance-trained athletes, high protein intake (~2.3 g/kg) during weight loss led to retention of FFM, but lower protein intake (~1 g/kg) during weight loss led to loss of FFM [30]. Collective literature suggests that a protein intake of 1.2–2.2 g/kg of absolute body mass is sufficient for athletes in positive EB [153,154,155,156]. However, a recent systematic review indicates a range of protein intake from 2.3–3.1 g/kg of FFM may be more appropriate for athletes undergoing ER with concurrent resistance exercise [137]. High protein intake is therefore a recommended dietary strategy for athletes to implement during IER, associated with increased satiety, a high thermic effect of feeding, and attenuated FFM losses. Protein supplements, protein dosing in close proximity to resistance exercise, and other dietary supplements including creatine and HMB may aid in FFM retention during ER, but a review of these topics is beyond the scope of this article. For further reading on these topics, we direct the reader to [137].

### 6.2. Carbohydrate Intake

#### 6.2.1. The Carbohydrate-Insulin Fat Loss Hypothesis

It has been suggested that a low carbohydrate diet may facilitate weight loss by providing a metabolic advantage [157]. This is based largely on the “carbohydrate-insulin” hypothesis that a lower carbohydrate intake will expedite lipolysis via diminished insulin activity. On the contrary, a recent meta-analysis concluded that a low carbohydrate diet was no more effective than a low fat diet in terms of weight loss [158]. Furthermore, a subtype of the low carbohydrate diet, the ketogenic diet, elevates levels of circulating ketones causing a physiological state known as nutritional ketosis. By restricting carbohydrates to less than 10% of total energy intake, ketosis has been proposed to cause a weight loss advantage by reducing insulin-mediated inhibition of lipolysis and enhancing fat oxidation through the utilization of ketone bodies [20]. However, a recent review has since determined that, when diet interventions match protein and energy intake between ketogenic and non-ketogenic conditions, no fat loss advantage to a ketogenic approach is observed [159]. Collectively, this suggests that the effectiveness of a weight loss diet is not dependent on ratios of carbohydrate and fat intake but instead the absolute amount of energy and protein ingested.

#### 6.2.2. Performance Considerations

Regardless of an athlete electing to take an intermittent or continuous approach to ER, a key concern is whether the composition of the diet is sufficient to support training and performance demands. Carbohydrate provides a versatile substrate for muscular work, supporting physical activity over a wide range of intensities due to its use by both anaerobic and aerobic pathways [160]. Higher carbohydrate intake may be preferable over higher intake of dietary fat by improving exercise efficiency, due to the greater yield of ATP per volume of oxygen deliverable to the mitochondria [161,162]. Additionally, evidence suggests that inadequate carbohydrate intake can impair both strength [163] and endurance performance [164]. Furthermore, the depletion of glycogen and blood glucose stores via low carbohydrate availability is associated with muscular fatigue, reduced work rate, and increased perception of effort [160]. Guidelines for carbohydrate intake recommend a range of 3–5 g/kg of absolute body weight per day for athletes competing in low intensity, skill-based sports and 6–10 g/kg/day for endurance sport athletes (1–3 h/day moderate to high intensity exercise) [165]. Higher carbohydrate intakes may also be preferable for bodybuilders, as a recent cross-sectional study recognised that, out of 51 competitors, those placing in the top five had greater carbohydrate intakes at the start of contest preparation (5.1 versus 3.7 g/kg of absolute body weight) than competitors who did not [143]. During ER, it may not be possible to reach the above carbohydrate recommendations with simultaneous high protein intake and low energy intake. Nonetheless, where protein targets are reached, it seems practicable for athletes undergoing IER to allocate a majority of their remaining energy allowance to carbohydrate.

#### 6.2.3. Refeeds

As discussed in a previous review, increased carbohydrate ingestion increases intramuscular glycogen storage, which may improve resistance exercise performance and recovery time, and allow an athlete to tolerate higher training volumes [166]. In a recent study, CrossFit athletes who increased their carbohydrate intake to 6–8 g/kg/day for three consecutive days demonstrated greater improvements in repetitions completed during a 12 min test compared to athletes with a carbohydrate intake <6 g/kg/day [167].

Leptin release also appears to be particularly responsive to increased carbohydrate intake [118,168]. An influx of carbohydrate on refeed days could also lead to a more pronounced muscle protein synthesis response if IER is applied in concert with resistance exercise through the insulin-mediated activation of the Akt/mTORC1 pathway [169]. Positive muscle protein balance could be further enhanced by insulin-mediated reductions in cortisol and muscle protein breakdown [170]. Although yet to be confirmed in the context of IER, it is possible that a more pronounced anabolic (or diminished catabolic) response to resistance exercise provided via carbohydrate refeeding could reduce FFM losses during ER. The current scientific literature suggests that preference should be given to increasing carbohydrate intake during refeed periods as opposed to increasing intake of protein or dietary fat.

As an additional theoretical rationale for carbohydrate-dominant refeeds, ER leads to an increase in whole body sensitivity to insulin and a concomitant alteration in both carbohydrate and lipid metabolism [171,172]. Specifically, ER has been demonstrated to cause suppression of postprandial and 24 h fat oxidation [173,174,175], with carbohydrate becoming the preferential fuel source in peripheral tissues for energy requirements [79,91,176]. Such a response increases the potential for dietary fat to be diverted toward adipose tissue as opposed to oxidative pathways. Preferential use of carbohydrate for energy needs while trafficking fat toward adipose is the most energetically efficient means of restoring depleted energy reserves, with the energy cost of depositing dietary fat (<2% of nutrient excess) being far less than the cost of depositing excess glucose (~25% of nutrient excess) via de novo lipogenesis [177]. Thus, low dietary fat/high carbohydrate refeeds may be superior to high fat refeeds in individuals undergoing ER with perturbed fuel metabolism, which favours the trafficking of a substantial amount of dietary fat toward lipid pools, in a system primed for very energetically efficient weight and fat regain.

### 6.3. Fat Intake

Sufficient dietary fat is an essential component of a balanced diet, facilitating the uptake of fat-soluble vitamins, supporting cell membrane structure, as well as providing energy [160]. Insufficient dietary fat intake may also lead to a decline in testosterone levels [178], potentially threatening the maintenance of FFM. However—as reviewed previously—research suggests that a high fat diet may impair an athlete’s ability to perform optimally during training and competitive efforts [179]. Studies using CER implemented with regular resistance exercise also demonstrate that higher-fat, lower-carbohydrate approaches [151,180] may be less effective than lower-fat, higher-carbohydrate approaches for preserving FFM [30,127]. Thus, if resistance exercise is implemented during IER then a higher carbohydrate intake in place of dietary fat may facilitate greater FFM preservation, likely through the maintenance of training volumes. Typically, dietary recommendations for athletes during ER are to maintain an adequate but lower end dietary fat intake (15–20% of energy intake) while emphasizing carbohydrate intake to fuel performance [1,137,160,165]. Of note, the above-listed nutritional considerations for the athlete are not exhaustive. For a more comprehensive examination of the implications of diets and body composition, readers should refer to the recently published position stand from the International Society of Sports Nutrition [21].

## 7. Summary

Athletes are known to have high levels of physical activity energy expenditure. Despite this, dietary interventions may be required to reduce body weight or body fat for maximising chances of competitive success. Ideally, this should be achieved via safe and effective nutritional strategies that minimise loss of lean tissues, health, and performance and reduce reliance on extreme or rapid weight loss practices. While yet to be demonstrated in athlete trials, IER is an effective means of reducing energy intake, body weight, and body fat. However, more importantly, some research suggests that IER may yield benefits over traditional CER by reducing some of the compensatory responses to ER, thereby increasing fat loss efficiency and reducing the likelihood of rapid weight regain. Furthermore—though yet to be confirmed—it is feasible that the implementation of high-carbohydrate refeeds may facilitate the maintenance of higher training volumes and augment the anabolic response following resistance training during these periods, potentially reducing FFM losses during ER. It is also possible that additional nutritional support provided by the coordination of refeed periods with a high volume or outcome-focused training phases/sessions may lead to improved performance. Finally, IER may be more acceptable to athletes as ER is only required during certain periods of the weight loss phase rather than the entire duration. While some advantages to an IER approach have been observed in non-athlete populations, we cannot yet confidently extrapolate these findings to athletes. Additionally, a number of the above-proposed benefits are still speculative.

Given the deficiency of research on IER in the context of athletes, optimal nonlinear dietary strategies are yet unknown. As such, there is a significant research opportunity for investigators interested in exploring various patterns of restriction (e.g., duration and level of energy intake for ER and refeed periods, ratios of ER to refeeds) and the manipulation of other dietary variables (e.g., macronutrition) to clarify how IER should be implemented in athlete populations as well as in the general public (if at all). Future investigation in the realm of IER should explore whether the recruitment of active individuals with healthy body composition, and the implementation of resistance exercise and optimised macronutrient intake would influence results achieved with IER. Nonetheless, we have provided a number of practical recommendations for an athlete wishing to implement IER in pursuit of his/her body weight or composition goals.

- Avoid severe IER and/or rapid weight loss. Severe ER may cause greater FFM losses than moderate ER, particularly in lean athletes. Severe ER may also adversely affect health and performance outcomes including reduced muscle strength, glycogen stores, and reflexes and increase the risk of injury due to fatigue and loss of FFM. It would be practicable for an athlete to adopt a moderate level of ER that encourages absolute body weight losses of 0.5–1% per week. Alternatively, an athlete may elect to reduce energy intake by a maximum of 35% relative to energy requirements for weight maintenance.
- Resistance exercise. Athletes implementing IER should be encouraged to partake in regimented resistance exercise as a means to attenuate FFM losses. Greater retention of FFM will likely minimise performance decrement during ER and may lead to greater fat loss efficiency by mitigating compensatory reductions in REE.
- Duration and ratios of ER and refeeds. With the limited human research available, a conservative practical recommendation is to alternate two weeks of moderate ER with two weeks in EB. Currently it is unknown whether this manipulation of energy intake is ideal for maximal fat loss and FFM retention or if additional arrangements of ER and refeeds may be superior.
- Coordinating refeed periods. It may be advantageous to synchronise intervals of EB with outcome-focused or high-volume training periods. This tactic may allow the athlete to perform optimally during training sessions by providing additional nutritional support and negating the adverse consequences of sustained, daily ER.
- High protein intake. High protein intakes may be beneficial to an athlete during IER by reducing FFM losses, providing greater satiety and increasing energy expenditure through the thermic effect of feeding. A daily protein intake range between 2.3 and 3.1 g/kg of lean body mass (which equates to approximately 2.0–2.6 g per kg of absolute body mass for an 80 kg athlete with 15% body fat) is likely an appropriate practical recommendation for athletes undergoing IER with concurrent resistance exercise.
- Emphasizing carbohydrate intake during refeeds. Although yet to be confirmed, it seems wise to place emphasis on increasing intake of carbohydrate during refeed periods opposed to increasing protein or fat. Elevated levels of leptin following carbohydrate feeding may cause stimulatory effects on energy expenditure and suppress appetite, leading to greater fat loss efficiency and easier diet adherence. Greater carbohydrate availability during refeed periods may also result in more pronounced anabolic responses when IER is applied in concert with resistance exercise, potentially reducing FFM losses during ER.
